# The Spatial Expansion and Ecological Footprint of Fisheries (1950 to Present)

**DOI:** 10.1371/journal.pone.0015143

**Published:** 2010-12-02

**Authors:** Wilf Swartz, Enric Sala, Sean Tracey, Reg Watson, Daniel Pauly

**Affiliations:** 1 Sea Around Us Project, Fisheries Center, University of British Columbia, British Columbia, Canada; 2 Centre d'Estudis Avançats de Blanes (CSIC), Blanes, Spain; 3 National Geographic Society, Washington, D. C., United States of America; 4 Marine Research Laboratories, Tasmanian Aquaculture and Fisheries Institute, University of Tasmania, Hobart, Tasmania, Australia; University of California San Diego, United States of America

## Abstract

Using estimates of the primary production required (PPR) to support fisheries catches (a measure of the footprint of fishing), we analyzed the geographical expansion of the global marine fisheries from 1950 to 2005. We used multiple threshold levels of PPR as percentage of local primary production to define ‘fisheries exploitation’ and applied them to the global dataset of spatially-explicit marine fisheries catches. This approach enabled us to assign exploitation status across a 0.5° latitude/longitude ocean grid system and trace the change in their status over the 56-year time period. This result highlights the global scale expansion in marine fisheries, from the coastal waters off North Atlantic and West Pacific to the waters in the Southern Hemisphere and into the high seas. The southward expansion of fisheries occurred at a rate of almost one degree latitude per year, with the greatest period of expansion occurring in the 1980s and early 1990s. By the mid 1990s, a third of the world's ocean, and two-thirds of continental shelves, were exploited at a level where PPR of fisheries exceed 10% of PP, leaving only unproductive waters of high seas, and relatively inaccessible waters in the Arctic and Antarctic as the last remaining ‘frontiers.’ The growth in marine fisheries catches for more than half a century was only made possible through exploitation of new fishing grounds. Their rapidly diminishing number indicates a global limit to growth and highlights the urgent need for a transition to sustainable fishing through reduction of PPR.

## Introduction

There is a wide realization that fisheries, similar to agriculture on land [1], has a tremendous impact on marine ecosystems and on the biodiversity embedded therein [2], [3]. This applies particularly to modern industrial fisheries, here defined as fisheries using craft powered by fossil fuel, which began in about 1880, when the first British steam trawlers were deployed. These quickly depleted the coastal population of flatfish and other bottom fish they were targeting, and they had to move offshore, gradually expanding into the entire northeastern Atlantic [4], [5]. A similar development was mirrored off New England, and along the coast of Japan, where local fish populations, already much reduced by operation conducted off sail-powered vessels (e.g., [6]), were strongly depleted.

The aftermath of the First and Second World War saw both a recovery of these stocks, and an increase in the sophistication of industrial vessels; which were equipped with diesel engine and increasingly sophisticated eco-locating equipment, and with refrigeration, enabling longer and longer trips. In 1950, the Food & Agriculture Organization of the United Nations [7] began issuing annual compendia of global fisheries statistics [8] which documented that global catches increased throughout the 1960s and 1970s, though the rate at which this increase proceeded slowly declined. In the late 1980s, global catches ceased to increase and peaked at 90 million t when account is taken of systematic over reporting of catches by China [9]. The slow decrease of about half million t per year which then ensued has not been reversed since [7], and is not likely to ever be [10].

This decrease occurred, essentially, because the rate at which new fish stocks (for example of deep sea fish; [11]) were accessed, from the late 1980s on, failed to compensate for the rate at which ‘traditional’ stocks were depleted. Moreover, the number of new stocks has been decreasing linearly over time [12]. This can be shown, e.g., using catch-status plots for different Large Marine Ecosystems [13], which account for the state of thousands of single-species stocks [14].

However, the global impact of fishing on the ecosystem, which includes species across the food chain from herbivores to top predators, cannot be fully assessed by the study of single-species catches. A more appropriate way of quantifying the expansion of and limits to fisheries is using the primary production required (PPR) to sustain catches – a metric of the ecological footprint of fishing. As defined by Pauly and Christensen [15], PPR allows direct comparison of the primary production required to generate a catch of a given (group of) species in a given time period (here: 1 year), and hence it allows for (indirect) comparisons between the catches of very different species of fish and invertebrates. Further, when the PPR of a given catch taken at a given locale is expressed as a fraction or percent of the primary production observed at that locale, we can use arbitrary thresholds of this fraction to define this locale as ‘exploited’, i.e., drawn into the scope of fisheries. Here we used different levels of “% PPR” (i.e., percentage of the primary production of the cells of a map of the global ocean) to quantify the expansion of fisheries since 1950 and extract the dominant patterns of this expansion.

## Results and Discussion

**Figure 1:**
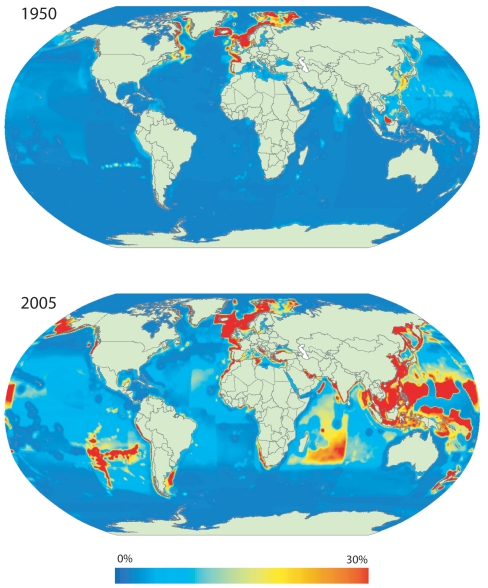
Primary production required (PPR) to sustain global marine fisheries landings expressed as percentage of local primary production (PP). Estimates of PPR, PP and PPR/PP computed per 0.5° latitude/longitude ocean cells. PPR estimates based on the *Sea Around Us* catch database (www.seaaroundus.org) and PP estimates derived from SeaWiFS's global ocean colour satellite data. The maps represent total annual landings for 1950 (top) and 2005 (bottom). Note that PP estimates are static and derived from the synoptic observation for 1998.

Most of the ecological footprint of fishing concentrated on the waters off the industrialized countries of North America and Europe, and off Japan in 1950, and have expanded to cover most of the world's productive waters by 2005. Figure 1 presents the spatial patterns of the proportion of the local primary production required to sustain the catch, for 1950 and 2005. These figures clearly demonstrate the expansion of fisheries, particularly of areas where the proportion of primary production exploited equal or exceed 30% (in red). The expansion is accompanied by the nearly five-fold increase in catch, from 19 million tonnes in 1950 (equivalent to 9 billion tonnes [wet weight] of primary production) to 87 million tonnes in 2005 (equivalent to 45 billion tonnes [wet weight] of primary production). In 2005, the footprint of one tonne of catch was, on average, 556 tonnes of PP (wet weight).

Some patterns in Figure 1 should be noted. First, the exploitation levels off the coast of East Africa in 2005 are likely to be underestimated due to underrepresentation of unreported catches in the region [16], [17]. Moreover, waters off the Pacific Island countries are known fishing grounds for tuna fisheries and reported to have a relatively high level of illegal and unreported catch [18].

**Figure 2:**
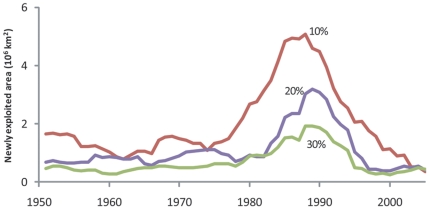
Time series of areas newly exploited by marine fisheries (1950–2005), expressed in km^2^. Newly exploited areas defined as regions where primary production required (PPR) to sustain reported fisheries landings exceeds the threshold percentage of local primary production (PP). Results based on three exploitation thresholds (10%, 20% and 30%) are presented.

**Figure 3:**
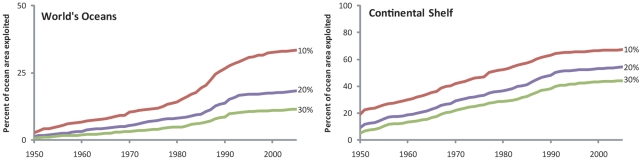
Time series of areas exploited by marine fisheries (1950–2005) expressed a percentage of the total ocean area. ‘Area exploited’ defined as regions where primary production required (PPR) to sustain reported fisheries landings exceeds the threshold percentage of local primary production (PP). Results based on three exploitation thresholds (10%, 20% and 30%), and for all marine areas (left) and continental shelf areas (i.e., up to 200 m in depth, right) are presented.

The rate of expansion can be illustrated by estimating the size of fishing grounds that become ‘newly exploited’ in each year. The 1980s to the mid 1990s were the period of greatest expansion (Figure 2), which corresponds to the period during which world catches began to stagnate, peaked and declined [9]. Similarly, Figure 3, which shows the cumulative area of the ocean that was exploited by fisheries based on multiple exploitation thresholds (10, 20 and 30%), highlights this accelerated expansion of the 1980s and the early 1990s. Comparison between the world ocean (left) and the continental shelves (coastal waters down to 200 m depth; right) shows that the accelerated expansion during this period was driven primarily through expansion into the open ocean. It should be noted that for both continental shelves and the world ocean, the pace of expansion slows down, because most commercially viable regions have been expanded into, leaving areas furthest away from fishing ports such as in the South Atlantic and the shelves off Antarctica.

**Figure 4:**
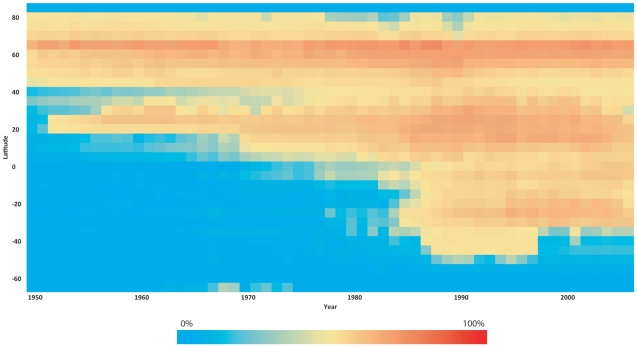
Time series of areas exploited by marine fisheries by latitude class, expressed as a percentage of the total ocean area. ‘Area exploited’ defined as regions where primary production required (PPR) to sustain reported fisheries landings is greater than 10% of local primary production (PP).

Figure 4 summarizes the direction of this expansion by presenting the time series of the proportion of the world ocean that has come to be exploited across latitudinal gradients. The figure shows that, even in the 1950s, the majority of the ocean surface in the North was already exploited and that, over time, an increasing proportion of the ocean in the South has become exploited. The waters near the poles are either covered in ice or away from fishing ports, rendering them unattractive, for now, to commercial exploitation.

**Figure 5:**
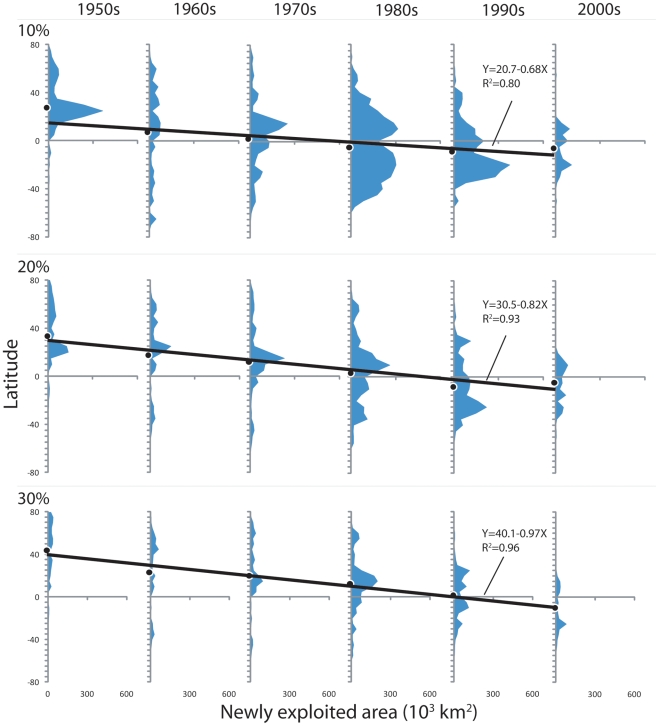
Newly exploited area (10^3^ km^2^) for each latitude class, averaged over each decade. Newly exploited area defined as ocean cells where primary production required to sustain fisheries catch exceeds the threshold percentage of primary production. Results based on three exploitation thresholds (10%, 20% and 30%) are presented. Black dots at the base of each histogram represent the mean latitude of the distribution. The dots for each exploitation threshold are fitted with a linear regression; jointly, they suggest the southward expansion of 0.7 to 0.9 degree per year.

Finally, Figure 5 quantifies the rate of this southward expansion by presenting the distributions of the areas of new exploitation for each decade. This expansion in marine fisheries was increasingly reliant on new fishing grounds in the South, with the means of these new fishing grounds shifting southward, on average, by about 0.8 degree per year. The northward deviations of the means from the regression line in the 2000s suggest that the expansion has run its course. This possibility is further confirmed the reduction in the size of newly exploited areas (i.e., areas under the curve) from 1990s to 2000s.

The expansion of the fisheries presented here can be viewed as an ecological footprint of the world fisheries. Ecological footprints are measured as the ratio between the productivity of the ecosystem and human consumption [19]. The standardization of fisheries catches into PPR enables footprints of various fisheries to be compared against the primary productivity of marine ecosystems.

The complexity and variability of fisheries and the marine ecosystems within which they are embedded therein make it difficult to define an across-the-board exploitation threshold of sustainability. An analysis of PPR across various Exclusive Economic Zones (EEZs) and Large Marine Ecosystems (LMEs) showed that fisheries exploitation can range from 1% in the Australian EEZ up to 80% in Icelandic EEZ, with varying impacts on the ecosystem ([20], and see contributions in [13]). The larger values are extraordinarily high compared with the 23.8% of potential net primary productivity humans appropriate on land [21].

Using PPR to calculate the loss of secondary production due to fishing, Coll et al. [22] showed that total catch per capita from Large Marine Ecosystems is at least twice the value estimated to ensure fishing at moderate sustainable levels. Chassot et al. [23] estimated that the primary production appropriated by current global fisheries is 17–112% higher than that appropriated by sustainable fisheries. In this study we also suggest that relatively low thresholds (between 10% and 30%) of PPR are sufficient to induce, and thus also track, expansion of fisheries.

These thresholds are more significant than they may seem, because the ecological impact of fishing depends on how much of the local primary production is available to sustain seafood production. For instance, only 41% of coastal phytoplankton is consumed by herbivores and moves up the food chain [24]. Therefore, the values of % PPR presented in this study are only a fraction of the actual proportion of primary production that is available for seafood production. In cases where fisheries capture more than 30% of local primary production (Figure 1), they may be capturing most of the PP available to fisheries. Further work is required to determine how much PP we are ‘overcapturing’. In other words, we need to estimate the proportion of primary production can be sustainably removed each year without compromising ecosystem integrity.

For our analysis, we assumed primary production to be constant over the study period, due to incomplete temporal coverage in the SeaWiFS dataset. While the level of primary production may have declined over the past 50 years concomitant with an observed reduction in the global chlorophyll concentration [25], the spatial patterns are thought to have been consistent at global scale [26]. The spatial patterns of expansion observed in our study should thus be independent of changes in global primary productivity, as evident by the similarities in the expansion patterns observed using three exploitation thresholds. However, our estimates of %PPR are likely conservative, and the footprint of fishing larger than reported here.

Nevertheless, the comparison with increase in agricultural production is startling. Tilman [1] observed that doubling of world agricultural production over the 35-year period, from 1961 to 1995, was accompanied by an increase of only 10% of the surface under cultivation. Over the same period, marine fisheries, which underwent a comparable 2.4-fold increase in catch (34 million tonnes to 83 million tonnes in catch weight or 17 billion tonnes to 44 billion tonnes in PPR, wet weight), required a nearly 4-fold increase in exploited area (when a 10% exploitation level is used as threshold).

Our results demonstrate that the growth in the world's marine fisheries over the past 56 years was driven through a sequential exploitation of new fishing grounds. Fisheries now cover a majority of the world's ocean, with areas of low productivity and distant waters as the final remaining ‘frontiers’. The decline of newly exploited areas since the late 1990s, which corresponds to a decline in global landings [7], implies that the era of great expansion has come to an end. With a limited room for expansion, and excessive appropriation of primary production in many regions, the only way toward sustainability of global fisheries goes through reduction of PPR.

## Materials and Methods

The analysis, which covers the period from 1950 to 2005, defines fisheries exploitation based on the primary production that is required to generate the catches of marine fisheries. The Primary Production Required (PPR), as proposed by Pauly and Christensen [15] is computed from: 
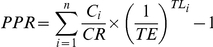
where *C_i_* is the catch of species *i*, *CR* is the conversion rate of wet weight to carbon, *TE* is the trophic transfer efficiency, *TL_i_* is the trophic level of species *i* and *n* is the number of species caught. We applied a 9∶1 ratio for *CR* and 10% for *TE* [15]. Species-specific trophic levels, usually derived from diet composition, i.e., stomach content data, were taken from FishBase (www.fishbase.org) for fishes and SeaLifeBase (www.sealifebase.org) for invertebrates.

Annual catch data were taken from the spatially disaggregated global catch database of the *Sea Around Us* project [27]. This online database (www.seaaroundus.org) is derived mainly from FAO global fisheries catch statistics, complemented by the statistics of various international and national agencies, and reconstructed datasets [27], [28]. These statistics, after harmonization, are disaggregated into a spatial grid system that breaks down world's ocean into 180,000 cells (0.5° latitude by 0.5° longitude) based on the geographical distribution of over 1500 commercially exploited fish and invertebrate taxa, using ancillary data such as the fishing agreements regulating foreign access to the Exclusive Economic Zones (EEZs) of maritime countries. Landing data were adjusted to account for discarded bycatch on the global estimates [29]. However no adjustment was made to account for regional or local variations in discards and other unreported catches.

Primary production estimates were derived using the model described by [30] which computes depth-integrated primary production based on chlorophyll pigment concentration based on SeaWiFS (www.seawifs.gsfc.nasa.gov) and photosynthetically active radiation as calculated in [31]. The estimates presented here pertain to 1998, which, for the purpose of our analysis, was assumed to be representative of the entire period.

Using the equation above and primary production estimates, we estimated for each year the proportion of primary production exploited in each of the 0.5° latitude/longitude ocean cells, defined as ‘exploited’ when the proportion of primary production exploited exceed a threshold level.
